# 5-HT1A Receptor Function Makes Wound Healing a Happier Process

**DOI:** 10.3389/fphar.2018.01406

**Published:** 2018-12-11

**Authors:** Alia Sadiq, Isabella Menchetti, Ahmed Shah, Marc G. Jeschke, Cassandra Belo, Wendolyn Carlos-Alcalde, Muhammad Qasim Hayat, Saeid Amini-Nik

**Affiliations:** ^1^Sunnybrook Research Institute, Toronto, ON, Canada; ^2^Atta-ur-Rahman School of Applied Biosciences (ASAB), National University of Sciences and Technology, Islamabad, Pakistan; ^3^Department of Laboratory Medicine and Pathobiology, University of Toronto, Toronto, ON, Canada; ^4^Division of Plastic Surgery, Department of Surgery, University of Toronto, Toronto, ON, Canada; ^5^Department of Immunology, University of Toronto, Toronto, ON, Canada; ^6^Ross Tilley Burn Centre, Sunnybrook Health Sciences Centre, Toronto, ON, Canada; ^7^Laboratory Medicine and Pathobiology, University of Toronto, Toronto, ON, Canada

**Keywords:** serotonin, 5-HT1A receptor, wound healing, 5-Ht1a receptor knockout mice model, skin regeneration

## Abstract

Skin wound healing is a multistage phenomenon that is regulated by cell–cell interplay and various factors. Endogenous serotonin is an important neurotransmitter and cytokine. Its interaction with the serotonin 1A receptor (5-HTR1A) delivers downstream cellular effects. The role of serotonin (5-hydroxytryptamine, 5-HT) and the 5-HT1A receptor has been established in the regeneration of tissues such as the liver and spinal motor neurons, prompting the investigation of the role of 5-HT1A receptor in skin healing. This study assessed the role of 5-HT1A receptor in excisional wound healing by employing an excisional punch biopsy model on 5-Ht1a receptor knockout mice. Post-harvest analysis revealed 5-Ht1a receptor knockout mice showed impaired skin healing, accompanied by a greater number of F4/80 macrophages, which prolongs the inflammatory phase of wound healing. To further unravel this phenomenon, we employed the 5-HT1A receptor agonist [(R)-(+)-8-Hydroxy-DPAT hydrobromide] as a topical cream treatment in an excisional punch biopsy model. The 5-HT1A receptor agonist treated group showed a smaller wound area, scar size, and improved neovascularization, which contributed to improve healing outcomes as compared to the control. Collectively, these findings revealed that serotonin and 5-HT1A receptor play an important role during the healing process. These findings may open new lines of investigation for the potential treatment alternatives to improve skin healing with minimal scarring.

## Introduction

The skin healing of full-thickness open wounds depends on a series of major cellular processes: epithelialization, cellular proliferation, migration, formation and contraction of granulation tissue. Cutaneous wound healing occurs via three main, yet overlapping phases: hemostasis and inflammation, proliferation, and remodeling. During the inflammatory phase, inflammatory cells (such as macrophages) release growth factors to enhance fibroblast and epithelial cell migration into the wound area (Amini-Nik et al., 2011, 2014; Bielefeld et al., 2011). Once the cells have migrated into the wound site, the proliferative phase begins, and these cells begin to reconstruct the extracellular matrix and aid in angiogenesis (Bielefeld et al., 2013; Shah and Amini-Nik, 2017). The remodeling phase primarily involves contraction of the wound and reorganization of the extracellular matrix, which is vital in determining the size and appearance of the resulting scar. The later phases are essential for maintaining tissue continuity. Pathological processes resulting in fibrosis are characterized by granulation tissue that results in excessive deposition of extracellular matrix and soft tissue deformation. In most cases, the wound healing process ends with scar formation (permanent deposition of connective tissue characterized by the presence of fibroblasts, extracellular matrix components, among which collagen is the most important component (Arno et al., 2014a; Amini-Nik et al., 2017; Amini-Nik, 2018). In chronic pathological cases, excessive extracellular matrix deposition may continue for several years; such processes are exhibited in hypertrophic scars and fibrotic lesions (Gabbiani, 2003).

Wound impairment occurs due to abnormalities at different stages of the healing process including diminished inflammatory response (Rodero and Khosrotehrani, 2010). Hypertrophic scarring occurs when the inflammatory phase of the healing process is perpetuated (Penn et al., 2012; Finnerty et al., 2016). During this phase, fibroblasts, keratinocytes and inflammatory cells migrate into the wound area and play an essential role in the healing mechanism. Macrophages are the primary mediators of this phase and are most abundant at 72 h post-injury. This inflammatory phase of wound healing is characterized by the release of various growth factors, cytokines, chemokines and neurotransmitters such as serotonin (DiPietro, 1995; Duerschmied and Bode, 2009). Serotonin or 5-hydroxytryptamine (5-HT) is a monoamine neurotransmitter (Stiedl et al., 2015). Serotonin’s role in potentiating the inflammatory response in the wounds of various organs has already been established, such as how 5-HTR antagonists have been associated with reduced hepatocellular regeneration (Lesurtel et al., 2006). Recently, there has been accumulating evidence that serotonin secreted by platelets and inflammatory cells plays an important role in cutaneous wound healing (Shah and Amini-Nik, 2017; Sadiq et al., 2018).

Endogenous serotoninergic stimulation via activated platelets is vital in activating macrophages and fibroblasts (Heldin and Westermark, 1999). Serotonin modulates macrophage polarization and regulates inflammation, and tissue repair via a large set of G-protein coupled receptors, 5-HTR 1-7 (de Las Casas-Engel and Corbi, 2014). Serotonin exerts its pleiotropic effects through different serotonergic receptors; for instance, 5-HT1 attenuates adenylyl cyclase resulting in decreased cAMP levels. Conversely, 5-HT2 enhances phosphoinositol (PI)-hydrolases, and 5-HT3 functions as a ligand-gated ion channel (Nordlind et al., 2008). Although 5-HTR3 and 5-HTR7 are also expressed in the skin, but 5-HT1 and 5-HT2 receptor subtypes are more widely expressed and have been the focus of most studies investigating the role of serotonin in various skin disorders (Lundeberg et al., 2002; Nordlind et al., 2006, 2008; Shah and Amini-Nik, 2017). There are several studies exploring the association between 5-HTR2A and 5-HTR2B antagonism in contributing to a diminished inflammatory response and fibrotic changes. Current research is still attempting to uncover the roles of different serotonin receptor subtypes (Ameisen et al., 1989; Lofdahl et al., 2016) but not particularly in the context of the role of the 5-HT1A receptor in skin wound healing. There is one study in which Buspirone has been implicated with the suppression of inflammatory effects (McAloon et al., 1995). Buspirone is a known 5-HT1A receptor agonist which acts as a full agonist at the somatodendritic 5-HT1A autoreceptor, and as a partial agonist at postsynaptic receptors (Eison and Temple, 1986; Cowen et al., 1994).

Serotonin is an emerging novel candidate as a mediator for wound healing. The potential role of serotonin through serotonergic receptors at various stages of wound healing has not been fully studied. Our previous study highlights the importance of endogenous serotonin in wound healing in the context of severe cutaneous thermal injury on scald burn mice (Sadiq et al., 2018). In this study, we investigated the specific mechanisms by which the serotonergic system enhances would healing through 5-HT1A receptor interaction. The presence of the 5-HT1A receptor in human dermal fibroblasts and keratinocytes has already been established (Slominski et al., 2003). This is further confirmed by our preliminary study showing the presence of 5-HT1A receptors in both human and murine keratinocytes and fibroblasts in comparison with other serotoninergic receptors (Supplementary Figure S1). This information along with the availability of 5-HT1A receptor knockout mice models, set the rationale behind the current study to investigate the role of serotonin in the wound healing process through its 5-HT1A receptor interaction. We have conducted a series of *in vitro* and *in vivo* experiments to better understand the role of the serotonin pathway during skin healing (Sadiq et al., 2018). Therefore, this study specifically investigates the role of 5-HT1A receptor in the context of cutaneous wound healing and scarring, which has not been investigated previously.

## Materials and Methods

### Excisional Wound Healing Model

#### Excisional Punch Biopsy Study in 5-Ht1a Receptor Knockout Mice Model

##### Animals

Young adult C57BL/6 mice and B6N (Cg)-Htr1a (6–8 weeks old, male, body weight 25 g) breeding pairs (heterozygote) were obtained from Jackson Laboratories (Bar Harbor, ME, United States). Several 5-Ht1a receptor knockout (homozygote, KO) adult mice were also obtained after successive breeding of B6N (Cg)-Htr1a breeding pairs, following all guidelines of the Animal Policy and Welfare Committee (Sunnybrook Research Institute and University of Toronto). B6N (Cg)-Htr1a Wild-type mice were the littermate of transgenic animal after breeding heterozygote animals together. The following procedures were carried out after approval from the above-mentioned committee.

(a) An excisional punch biopsy procedure was conducted on the B6N (Cg)-Htr1a knockout (KO) (*n* = 10) group and wild-type (WT) mice groups (*n* = 10) to determine the role of the 5-HT1A receptor in skin injuries. The mice were anesthetized with isoflurane (2–3%) and their body weights were recorded. The dorsal surface of the mouse was shaved and sterilized. Buprenorphine was administered via intra-peritoneal injection and a 4 mm diameter full-thickness skin excisional wound was created through a punch biopsy (4 punches per mouse). The mice were monitored until they were fully awake and later returned to the animal facility. Wound healing was monitored and detailed observations were recorded until harvest day (1-week post wound). The mice were then anesthetized with isoflurane and euthanized via cervical dislocation at the end of the study and processed for wound harvesting.

(b) A second excisional punch biopsy study was carried out to investigate the effect of 5-HT1A receptor agonist on wound healing. 5-HT1A receptor agonist “8-Hydroxy-DPAT hydrobromide” (Hoyer et al., 1994) was used in this study in the form of a topical cream. The study was conducted on C57BL/6 mice, divided into an agonist treatment group (*n* = 5) and a control group (*n* = 5). The cream formulation (1%) was prepared by using the Transderma PLO Kit (Transderma Pharmaceuticals Inc., Canada) according to the manual’s instructions for topical application. The excisional punch biopsy procedure was conducted via the methods described above. Treatment: Mice wounds were treated topically with the 1% cream formulation, twice a day for 1-week post-injury. The control group was treated with vehicle cream. Wound healing was then monitored and recorded until harvest day (as previously explained).

(c) Excisional punch biopsy experimentation was extended further to investigate the role of the 5-HT1A receptor agonist in neovascularization during skin wound healing. The C57BL/6 mice group (*n* = 10 mice) received a 4 mm punch biopsy wound (decribed previously) and were then divided into two categories. In the first category, an agonist (*n* = 5 mice) and control group (*n* = 5 mice) study continued for 5-days, while in the second, the same study continued for 7-days. Both treatment groups received topical cream (preparation mentioned above) treatment twice a day and wound healing was monitored until respective harvest time (as mentioned previously) (Supplementary Figure S2) (Ansell et al., 2014).

##### Wound analysis

Wound measurements were taken and wound closure was examined during each study. In the second study, mice were injected intraperitoneally with 20 μl/g body weight of BrdU labeling reagent (Roche Diagnostics GmbH, Mannheim, Germany) 24 h before harvesting. Mice were sacrificed at 5- and 7-day time points. Skin biopsies including the wound/scar site and 2 mm of satellite skin were harvested for further analysis (Mori et al., 2004).

##### Histologic analysis

Histological assessment was carried out on sections derived from the widest part of the wound, in the wound center. Thus, only the most completely disrupted part of the wound was considered for healing assessment. By implementing this strategy, distinct changes in the wound-healing process can be assessed and easily reproduced (Braiman-Wiksman et al., 2007). Skin tissue samples were initially fixed in buffered formalin (10%, overnight at room temperature), then preserved in 70% ethanol and processed in paraffin. Skin wound specimens were cut into thin sections (5 μm) and at both the center of the wound and on each side, eliciting a cross-section through the entire wound and satellite area. These wound sections were then subjected to trichrome and immunohistochemical staining and respective wound analysis (Ansell et al., 2014).

#### Masson’s Trichrome Staining of Post-harvested Skin Wound Sections

Paraffin-embedded slides were deparaffinized with citrosol, followed by rehydration through 100, 95, 70, and 50% ethanol to water. Slides were placed in Bouin’s solution (26367-01; EMS, Hatfield, PA, United States) overnight at room temperature and washed. A hematoxylin stain (HHS16; Sigma-Aldrich, St. Louis, MO, United States) and Biebrich scarlet-acid fuchsin solution were applied sequentially for 10 min. After each stain, the slides were washed. Next, slides were differentiated in phosphomolybdic–tungstic acid for 15 min and transferred into aniline blue for 5 min. All slides were rinsed appropriately and differentiated in 1% acetic acid for 2 min. Slides were then dehydrated with 95% ethanol and absolute ethanol followed by clearing in citrosol. The slides were mounted with xylene-based liquid mounting media (Triangle Biomedical Sciences, Durham, NC, United States). All trichrome reagents were purchased from EMS (Hatfield, PA, United States) and freshly prepared. After completing the staining process, the slides were photographed at 5, 20, and 40× magnification using a light microscope (Zeiss Axiovert 200, Germany). Quantification was carried out to measure the epidermal thickness (micrometers), wound cellularity (total cell count) at the wound center/dermal area, wound size (total wound area in μm^2^) and wound length (micrometers) from left and right margins. All analysis was done blindly between the samples (5 images/wound sample). During the analytic process, the names of the images were all substituted for numbers, ensuring the examiner was blinded to the treatment groups. Masson’s Trichrome staining shows the following changes: nuclei stained black, cytoplasm, muscles and erythrocytes stained red and collagen stained blue (Braiman-Wiksman et al., 2007; Ansell et al., 2014).

#### Immunohistochemistry

Paraffin-embedded skin tissue slides were subjected to immunohistochemical staining. First, the slides were deparaffinized with xylene and then rehydrated through 100, 95, 70 and 50% ethanol to water. Antigen decloaker (1X; Biocare Medical, Concord, CA, United States) was added to the slides in a preheated decloaking chamber for 4 min at 110°C. For BrdU staining, samples were denatured with 1.5 M HCl for 30 min at 37°C and neutralized with 0.1 M borate and buffered twice for 5 min. Samples were blocked with 3% H_2_O_2_ for 10 min and then washed with a washing buffer (0.05 M Tris–HCl, 0.15 M NaCl, and 0.05% Tween 20 in deionized water). Primary antibodies, mouse monoclonal anti-BrdU (1:200; Cell Signaling, Beverly, MA, United States), mouse monoclonal anti-msASM (1:400; eBiosciences), anti- msKi67 (1:400; cell signaling), rat monoclonal anti-F4/80 (1:200 AbD Serotec), CD31 (1:250, catalog number NBP1-71663, Novus Biologicals, United States) were diluted in PBS and incubated at room temperature for 1 h. Firstly, the slides were incubated for 15 min with an MACH3 mouse probe (Biocare Medical). The slides were then washed with PBS, incubated a second time with an MACH3 rabbit or mouse horseradish peroxidase polymer, and washed again with PBS when finished. The betazoid diaminobenzidine chromogen kit (Biocare Medical) was mixed and added onto the slides for 5 min, and the reaction was terminated with running water. Later, nuclear staining was carried out with hematoxylin for 30 s, followed by differentiation with 3 dips in 1.5% acid alcohol and bluing in 0.1% sodium bicarbonate for 10 s. Sections were dehydrated with 95% and absolute ethanol to citrosol and mounted with xylene-based liquid mounting media. Images were acquired using a Zeiss Axiovert 200 light microscope at 10 and 40× magnification. The positive expression for ASM, Ki67, BrdU, CD31 and F4/80 staining was quantified by ImageJ software by a blind observer (Weller et al., 2006; Arno et al., 2014b).

### *In vitro* Experiments

#### Drugs, Reagents and Culture Media

(R) – (+)-8-hydroxy-DPAT hydrobromide as 5-HTR1A agonist (Tocris Bioscience, Canada). Tranderma PLO Kits (Transderma Pharmaceuticals Inc., Canada), Buprenorphine (Sigma Aldrich^®^ Inc.). TRIzol Plus RNA Purification Kit, cDNA Reverse Transcription kit, DreamTaq PCR Master Mix -2X (Thermo Fisher Scientific^®^ Inc.). SYBR safe (Invitrogen). Fibroblast culture medium Dulbecco’s modified Eagle’s medium (DMEM) (containing 10% fetal bovine serum and 1% antibiotic–antimycotic solution) and supplements (Life Technologies). Keratinocyte culture medium EpiLife^®^ Medium, human Keratinocyte Growth Supplement (HKGS) and Trypsin (Gibco^TM^ Burlington, ON, Canada). Cell culture media was protected from light and preserved at 2–8°C for experimental use.

#### Human Skin Tissue Sampling

The normal human skin tissues were obtained from healthy male and female donors who underwent plastic surgery procedures at the Ross Tilley Burn Centre, Sunnybrook Health Sciences Centre, Toronto, ON, Canada. Pregnant females were excluded. The collection of skin tissue samples was approved by the Institutional Ethics Review Board (Declaration of Helsinki Principles, Academic Health Sciences Network and University of Toronto-affiliated Sunnybrook Research Institute and Sunnybrook Health Sciences Centre, Toronto, Canada). Informed, signed consent was obtained from the patient. After collection, skin tissue samples were processed aseptically. Samples were cut into small pieces and cleaned to remove the hypodermis and any fat. Some fresh skin pieces were used for primary cell culture, and the rest of the pieces were snap frozen at -80°C for further *in vitro* experiments. Mice skin samples were also processed according to the above-mentioned protocol (Arno et al., 2014a).

### Cell Culture

Human primary skin fibroblasts were obtained from normal human skin tissue samples. Small tissue sizes taken from normal skin tissue specimens were defined as the explant. The explant was cultured in small petri plates (37°C in a humidified atmosphere with 5% carbon dioxide) in fibroblast culture medium (DMEM) to obtain primary fibroblast cultures. The primary cultures were sub-cultured in petri plates (density of 3,200 cells/cm^2^) for 1 week and later subjected to further passaging. The primary culture (70% confluency) was subjected to trypsinization (0.05% trypsin) and sub-cultured further (4,500 cells/cm^2^) into flasks (Corning^®^ cell culture flasks 75 cm^2^). The primary mouse fibroblasts obtained from the 5-Ht1a receptor knockout and wild-type mice were cultured and passaged under the same protocol separately (Galban et al., 2013). Human epidermal keratinocytes, neonatal (HEKn), and murine keratinocytes were obtained from in-house cell cultures stored in liquid nitrogen and sub-cultured in keratinocyte culture medium EpiLife^®^ Medium and human Keratinocyte Growth Supplement (HKGS) in flasks (Li et al., 2017). All cultures were trypsinized until confluent and then processed for isolation of total RNA and respective *in vitro* experiments.

### RNA Extraction and cDNA Preparation

Total RNA was isolated from human skin tissue, fibroblasts, neonatal epidermal keratinocytes (HEKn), murine fibroblasts and keratinocytes by using the Trizol RNA isolation kit. Isolated RNA samples were purified by using the RNAs purification kit. RNA quality was evaluated by using a Nano drop spectrophotometer (Thermo Fisher Scientific). cDNA was prepared from all samples by using the cDNA Reverse Transcription kit. The RT-Thermal cycler (Thermo Fisher Scientific) program consisted of step 1 at 25°C for 10 min, step 2 at 37°C for 120 min, step 3 at 85°C for 5 min and step 4 at 4°C. All cDNA samples were used for PCR and any remaining samples were stored at -20°C for future use (Slominski et al., 2003).

### Polymerase Chain Reaction (PCR)

The expression of serotonin receptor genes (5-HTR1A, 1B, 2A, 2B and 7) was evaluated by using reverse transcription (RT)-PCR for all the cDNA samples. Human serotonin receptor 1A was amplified by 5-HTR1A-F (5′AGGGCAACAACACCACATCAC3′) and Hu-5-HTR1AR (5′GACCGCCAAAGAGCCAATAAG3′). Serotonin receptor 1B was amplified by Hu-5-HTR1B-F (5′GTGGGTCTTCTCCATCTCTATC 3′) and Hu-5-HTR1B-R (5′ GTCCTGTTGGGCGTCTGTTTC 3′). Serotonin receptor 2A was amplified by Hu-5-HTR2A-F (5′CAGCCGCTTCAACTCCAGAAC 3′) and Hu-5-HTR2A-R (5′AGCCGATCAGGACAAAGTTATC 3′). Seroton in receptor 2B was amplified by Hu-5-HTR2B-F (5′TGGGCAGCTCTTCTGATACTCATG 3′) and Hu-5-HTR2B-R (5′ AGGTGTGAAGAAGGCAGCCAGTG 3′). Serotonin receptor 7 was amplified by Hu-5-HTR7-F (5′ GAACAGATCAACTACGGCAGAGTC 3′) and Hu-5-HTR7-R (5′ GACATGGGGATATAAAATGCCACTG 3′). Mouse serotonin receptor 1a was amplified by Ms-5-Htr1a-F (5′AAGGAAGTGTGGGTGTGAGG 3′) and Ms-5-Htr1a-R (5′TCTGCGAAATTTGTGTGAGC 3′). GAPDH gene expression was tested in all samples to check the integrity of the DNA. GAPDH expression was amplified in human and mice samples by using the following primers: Hu-GAPDH-F (5′ACAGTCAGCCGCATCTTCTT 3′), Hu-GAPDH-R (5′ ACGACCAAATCCGTTGACTC 3′), Ms-gapdh-F (5′GCCGCCTGGAGAAACCTGCCAAGT 3′) and Ms-gapdh-R (5′ TATTCAAGAGAGTAGGGAGGGCTC 3′). The PCR reaction mixture (25 μL) was prepared using DreamTaq PCR Master Mix (2X). Serotonin receptors 1A, 1B, and 2A were amplified through 30 cycles: 30 s at 98°C, 30 s at 56°C, 40 s at 72°C, and the final 7 min at 72°C. Serotonin receptor 2B and 7 were amplified by a PCR program (Thermal cycler, Thermo Fisher Scientific) that was set at 35 cycles; 30 s at 94°C, 40 s at 63°C, 45 s at 72°C, and the final 7 min at 72°C. All amplified products were resolved as single bands on an agarose gel (2%) through electrophoresis and visualized with SYBR safe fluorescence in a gel documentation system (Thermo Fisher Scientific) (Slominski et al., 2003).

### Apoptosis Assay

Mouse fibroblasts (5-Ht1a receptor knockout and wild-type) were separately cultured (5,000 cells/well) in 96-well plates and grown in fibroblast culture medium. After 24 h of incubation, the media was replaced with fresh media and incubated further for another 12 h. Caspase activity was assessed by using the Luminogenic caspase-3/7 substrate assay (Caspase-Glo^®^ 3/7 Assay, Promega). Relative fluorescence (RFU) was observed by using a microplate reader (Thermo Fisher Scientific) (Schweizer and Winter, 1983).

### Viability Assay

Mouse fibroblasts (5- Ht1a receptor knockout and wild-type) were cultured (5,000 cells per well) in 96-well plates and incubated for 24 h. Effects on cellular viability were observed by using the CellTiter-Glo^®^ Luminescent cell viability assay kit (Promega, Madison, WI, United States) following the manual’s instructions. The absorbance was measured at 550 nm on a microplate reader (Thermo Fisher Scientific). Results were analyzed and presented as a Relative Luminescent Unit (RLU) (Gutowska-Owsiak et al., 2014).

### Proliferation Assay

Mouse fibroblasts (5-Ht1a receptor knockout and wild-type) were cultured separately (6000 cells/cm^2^) in 8-chamber glass culture slides, until 50–60% confluent. Culture media was then replaced by fresh media. Bromodeoxyuridine, BrdU (1:200) was added to each chamber and kept for an additional 24 h of incubation (Singer and Clark, 1999). For the immunofluorescence analysis, first, cultures were washed with PBS (phosphate-buffered saline) and fixed with Paraformaldehyde 4% (Alfa Aesar. Karlsruhe. Germany) for 15 min. Cells were then washed again with PBS and covered in PBST (containing 0.5% Triton X-100 solution and PBS) for 10 min for permeabilization. After further washing, the fixed cells underwent blocking (30 min) with bovine serum albumin (BSA, 1%) in PBST. The primary monoclonal antibody, mBrdU, ratio 1:200 (cell signaling) was added and left for incubation overnight at 4°C. Afterward, the cells were washed again with PBS, a secondary antibody 1:500 (Anti-mouse, Alexa Fluor-488, Life Technologies) was added on the slides, and they were kept for incubation in the dark at room temperature for 1 h. Following incubation, slides were washed with PBS and mounted with Vectashield, containing DAPI (Vector Laboratories, United States). After staining, cells were observed and photographed at 20× magnification by using the axiovert fluorescent system (Zeiss, Germany). Results are presented as a mean percentage of BrdU +ve cells with 95% confidence intervals (Sinno and Prakash, 2013).

### Cell Migration (Scratch Wound Assay)

To assess the effects of the 5-HT1A receptor knockout on cell migration, 5-Ht1a receptor knockout, and wild-type mouse fibroblasts were cultured for 24 h (20,000 cells per slide) in culture glass slides (Thermo Scientific. Lab-Tek chamber slides). Scratches were made by using a 1000 μl pipette tip and followed by a PBS wash. Old media was then replaced with fresh media and incubated for a further 24 h (Gutowska-Owsiak et al., 2014). Next, 4% Paraformaldehyde was used to fix the cultured cells for immunofluorescence staining. Phalloidin antibody (1:30) conjugated to fluorescein isothiocyanate (Invitrogen) was added into the cell culture and incubated for 1 h in a blocking solution. Cells were washed with PBS and subjected for mounting with Vectashield, containing DAPI. Imaging at 10× magnification was carried out by using a laser scanning microscope (META 510 confocal microscope, Zeiss, Germany) and quantified using ImageJ (National Institutes of Health, Bethesda, MD, United States) (Arno et al., 2014b). Results were presented as the comparative width of the scratch zone in micrometers (μm) in knockout (KO) and wild-type (WT) mouse fibroblast cultures after 24 h.

### Statistical Analysis

All experiments were performed three times. Data was statistically described in terms of mean number of cells ± standard deviation (±SD), 95% confidence interval and percentage (%) when appropriate. The comparative statistical analysis was carried out by using the student’s *t*-test. Correlation between various variables was done using Pearson correlation equation for non-normal variables. The entirety of the data was analyzed by using a two-way ANOVA and Tukey *post hoc* tests. Significance levels were set at ^∗^*P* < 0.05 and ^∗∗^*P* < 0.01. All statistical analysis was done using Microsoft Excel version 8.

## Results

### 5-HT1A Receptor Expression in Human and Murine Fibroblasts and Keratinocytes

As a preliminary study, the expression of five serotonin receptors (5-HT1A, 1B, 2A, 2B and 7) in human skin tissue, human and murine fibroblasts, and keratinocytes, has been evaluated. Expression analysis, using gel electrophoresis of PCR products revealed that the 5-HT1A receptor is abundantly present in all skin samples (Supplementary Figure S1). Isolated human and murine fibroblasts and keratinocytes showed that these two cell types express the 5-HT1A receptor (Supplementary Figure S1), suggesting that this receptor may be important for skin homeostasis and healing. We carried out further experiments in mice to further understand the role of the 5-HT1A receptor during skin healing.

### Deficient Skin Healing in 5-Ht1a Receptor KO Mice

**FIGURE 1:**
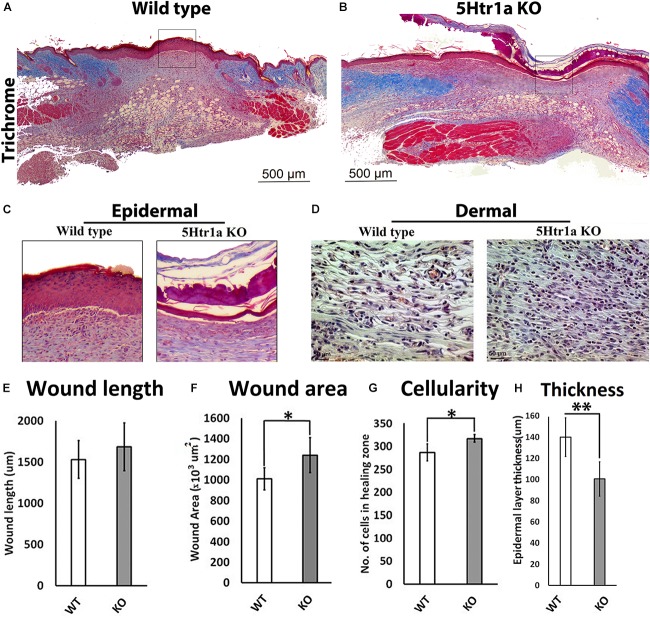
Excisional wound healing in 5-Ht1a receptor KO mice (excisional punch biopsy model). The wound tissue sections were prepared 7-days after wounding and Masson’s trichrome staining was performed. Scale bars represented at 500 μm **(A,B)**. Comparative epidermal thickness (μm) between WT and KO group **(C,H)**. Comparative wound length (μm) in WT and KO group **(E)**. Wound area (×10^3^ μm^2^) is comparatively bigger in KO group **(F)**, (*n* = 4 wound images per specimen). Wound cellularity and total cell count (number of cells in wound zone) is comparatively high in KO group **(D,G)**, images represented at 50 μm scale bar (*n* = 4 images per specimen). Results were presented as mean ± 95 CI (confidence interval). Two-way ANOVA was performed and significance levels were set at ^∗^*P* < 0.05, ^∗∗^*P* < 0.01.

**FIGURE 2:**
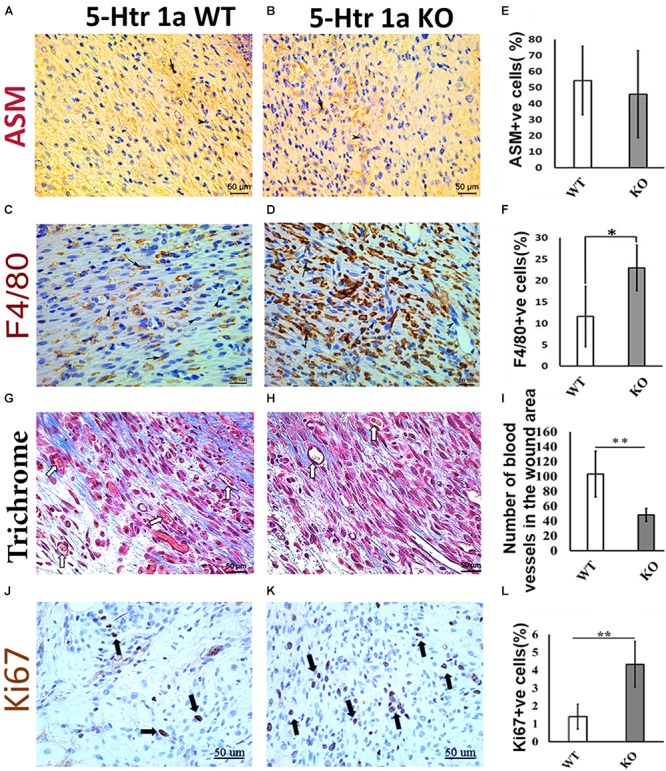
Comparative histochemical and immunohistochemical analysis for ASM, F4/80, Ki67 expression and neovascularization in 5-Ht1a receptor KO and Wild-type mice (excisional punch biopsy model). Dermal fibroblasts in wound area showed non-significant ASM expression in both group WT and KO, **(A,B,E)**. Macrophage count (percentage of F4/80 +ve cells) is high in the wound area of the KO group as compared to WT **(C,D,F)**. Trichrome staining showed mean number of blood vessels in wound area of the KO mice group is lower than the WT mice group **(G–I)**. Expression of Ki67 +ve cell percentage is higher in the KO mice group as compared to WT **(J–L)** (*n* = 4 images per specimen). Arrow indicates cells expressing markers and arrow heads indicate absence of marker expression. All images were presented at 50 μm scale bar. Results presented as mean ± 95 CI (confidence interval). Two-way ANOVA was performed and significance levels were set at ^∗^*P* < 0.05, ^∗∗^*P* < 0.01.

To understand the role of 5-HT1A receptor during skin healing, an excisional wound healing study (excisional punch biopsy) was conducted in 5-Ht1a receptor knockout mice and compared with wild-type mice. Post-harvest wound analysis showed that 5-Ht1a receptor knockout mice (KO) exhibited poor wound healing compared to the wild-type group (WT) (Figures 1A,B). Newly formed epidermal layers over the wound area were significantly thinner in the KO animals (100.5 ± 49.7 μm < 140 ± 29.4 μm, respectively) (Figures 1C,H). While no change in wound length was observed (Figure 1E), the total wound area was bigger in the KO animals compared to the WT group (1,240.24 × 10^3^ μm^2^ ± 376 > 1,009.38 × 10^3^ μm^2^± 198, respectively) (Figure 1F). The larger wound area in the KO group also had a significantly high wound cellularity (number of cells 317 ± 22.9 > 286.6 ± 30.2, respectively) (Figures 1D,G). This was further supported by a significantly higher number of Ki67 +ve cells in the KO animals (percentage of Ki67 +ve cell = 4.33 ± 2.90 > 1.4 ± 1.0, respectively) as compared to the WT group (Figures 2J–L). This raises the possibility that the serotonin pathway involving 5-HT1A receptor, regulates the reconstruction of the skin during the healing process, leading to enhanced healing. When knocked out ubiquitously, the wounds showed delayed maturation, and active proliferation within the granulation tissue, leading to a bigger wound area.

### 5-Htr1a KO Animals Show Characteristics of Prolonged Skin Healing in Comparison With Control Animals

To verify whether the larger wound area in the KO animals is due to the delayed myofibroblastic phenotype in granulation tissue, we subjected the harvested wounds to immunohistochemical analysis. Immunohistochemistry using α-smooth muscle actin (ASM) antibody revealed no significant difference in myofibroblast abundance between the KO and WT mice (45.8 ± 27.2% < 54.3 ± 21.3%) (Figures 2A,B,E). As the transient myofibroblastic phenotype contributes in wound contraction, these results suggest that 5-HTR1A does not affect the transition of fibroblasts into myofibroblasts which results in a comparable wound length in both groups. To understand why the total number of cells increased in the KO animals (accompanied with a higher percentage of Ki67 +ve cells), we stained the wound sections through immunohistochemistry to aid in macrophage quantification (F4/80). A significantly higher number of macrophages was seen in the granulation tissue of the KO aniamls compared to the WT group (23.76 ± 3.1% > 11.56 ± 5.6%, respectively) (Figures 2C,D,F). Moreover, the number of blood vessels was higher in the WT animals (WT = 103 ± 30.8 > KO = 48 ± 8.8) (Figures 2G–I). This finding suggests that wounds in the WT animals are further along in the healing process, past the inflammatory phase (fewer macrophages), and entering into the later proliferation and maturation phase where a higher number of blood vessels is expected.

### 5-Ht1a Receptor KO Murine Fibroblast Showed Deficient Migration in Scratch Wound Assay

**FIGURE 3:**
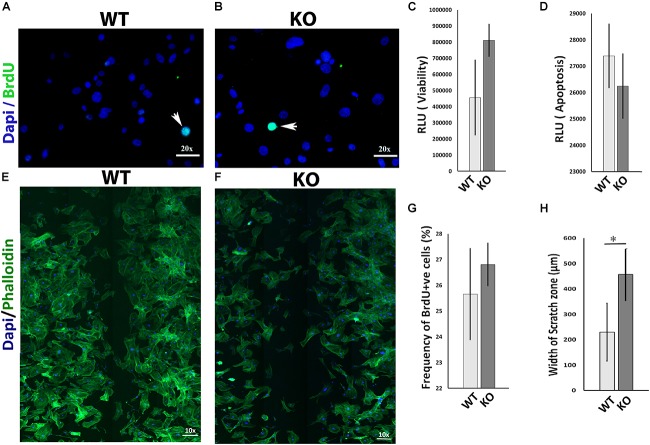
*In vitro* comparative study for cell viability, apoptosis, proliferation, and migration between 5-Ht1a receptor Knock out and Wild-type mouse fibroblast cultures. Comparative proliferation **(A,B,G)**, viability **(C)** and apoptosis **(D)** in KO and WT fibroblasts (*n* = 5 images per sample). Migration assay staining with DAPI and Phalloidin **(E,F)**. Scale bar represents the image magnification. Comparative fibroblast migration (KO vs. WT) was measured in term of width of the scratch zone (μm) from Time 0 to Time 18 h **(H)**. Graphical results were presented as mean ± 95 CI (confidence interval). All experiments were performed three times. Two-way ANOVA was performed and significance levels were set at ^∗^*P* < 0.05, ^∗∗^*P* < 0.01.

The migration of mesenchymal cells such as fibroblasts into the wound bed is an important determinant for normal wound healing (Bainbridge, 2013; Amini-Nik et al., 2014; Lee et al., 2016). To investigate whether the 5-HT1A receptor activity regulates wound healing partly through fibroblast behavior, we examined several ways in which it could influence fibroblasts *in vitro*. 5-Ht1a receptor KO mouse fibroblasts were isolated and utilized in a series of *in vitro* experiments. Our data revealed that lacking the 5-HT1A receptor does not affect fibroblast viability, apoptosis, and proliferation *in vitro* (Figures 3A–D,G) but significantly inhibits cell migration. Through an *in vitro* scratch wound assay, unlike fibroblasts isolated from the WT animals, KO fibroblasts showed deficient migration (Figures 3E,F,H). This data proposes that the 5-HT1A receptor contributes to the regulating of skin healing, partly through its affects on fibroblast migration.

### 5-HT1A Receptor Agonist Improved Skin Healing

**FIGURE 4:**
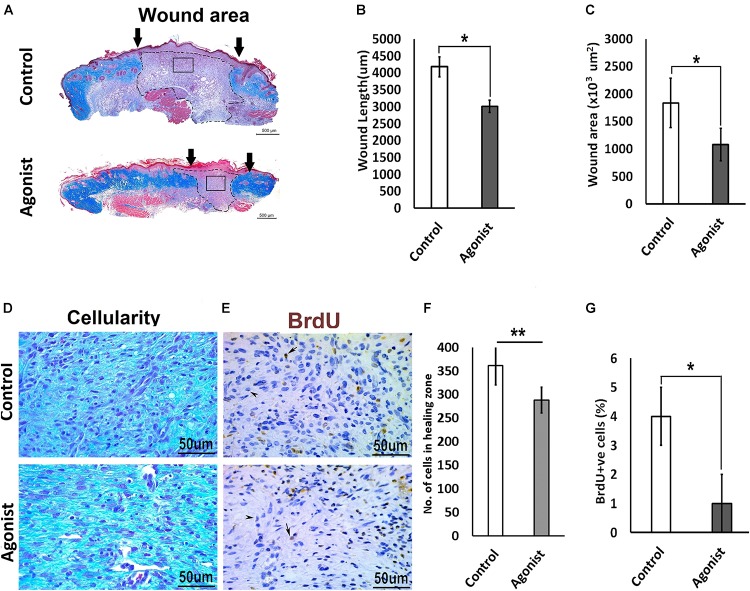
Comparative excisional wound healing in control and 5-HTR1A agonist treated group (excisional punch biopsy model). The wound tissue sections were prepared 7-days after wounding and Masson’s trichrome was staining performed. Scale bars represented at 500 μm **(A)**. Wound length (μm) decreased in 5-HTR1A agonist treated mice group **(B)**. Significantly reduced wound area (×10^3^ μm^2^) shown in 5-HTR1A agonist treated group **(C)** (*n* = 2 wound images per specimen). Wound cellularity significantly decreased in 5-HTR1A agonist treated group **(D)**, image scale bars represented at 50 μm. Comparative wound cellularity was expressed as percentage number of cell count (*n* = 2 wound images per specimen) **(F)**. Comparative percentage BrdU +ve cells between agonist and control, image scale bars represented at 50 μm **(E,G)**, (*n* = 4 images per specimen). Results were presented as mean ± 95 CI (confidence interval). Two-way ANOVA was performed and significance levels were set at ^∗^*P* < 0.05, ^∗∗^*P* < 0.01.

To investigate whether the activation of the serotonin pathway through the 5-HT1A receptor enhances wound healing, we treated wounds topically with the receptor agonist (8-Hydroxy-DPAT hydrobromide) *in vivo*, in wild-type mice (C57BL/6 background). Wounds treated with 5-HT1A receptor agonist cream showed a significantly smaller wound scar size (wound length, 3011.2 ± 533 μm < 4177 ± 833 μm; wound area, 1078 ± 455 × 10^3^ μm^2^ < 1834.7 ± 690 × 10^3^ μm^2^, respectively) (Figures 4A–C) accompanied with lower cellularity (307.75 ± 63.8 < 475 ± 97.7, respectively) (Figures 4D,F) compared to the vehicle-treated control wounds. This finding suggests that 5-HTR1A agonist treatment most-likely regulates cell density through modulation of cell viability or cell motility. To confirm this, we subjected the animals to BrdU before harvesting the wounds. A significantly lower proliferation activity was observed in the agonist-treated group (percentage of BrdU +ve cells, 1 ± 0.034% > 4 ± 0.03%) compared to the control (Figures 4E,G). In addition to the data from the KO mice experiments, these results indicate that the 5-HTR1A-agonist may enhance fibroblast migration, resulting in a faster reconstitution of the dermis. Therefore, the reconstructed skin shows a lower proliferation activity (fewer BrdU +ve cells) in comparison with the vehicle-treated wounds.

### 5-HT1A Receptor Agonist Enhances Neovascularization and Accelerates Wound Healing

**FIGURE 5:**
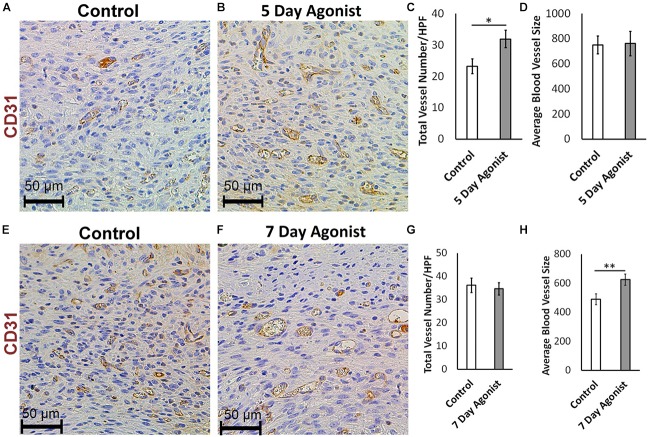
5-HTR1A agonist enhances neovascularization during skin wound healing (excisional punch biopsy model). CD31+ endothelial cell immunohistochemical staining at day 5 **(A,B)** and day 7 **(E,F)**, image scale bars represented at 50 μm. 5-HTR1A agonist treatment showed a significant increase in blood vessel count at day 5 **(C)** compared to day 7 **(G)**. Size of the blood vessels (average size of blood vessels in μm^2^) in the agonist group were larger at day 7 **(H)** compared to day 5 **(D)**. Results were presented as mean ± SEM (*n* = 5 images per wound). Two-way ANOVA was performed and significance levels were set at ^∗^*P* < 0.05, ^∗∗^*P* < 0.01.

Evaluating neovascularization at different time points during healing is an effective method for understanding the wound healing process in greater depth. CD31 immunohistochemical staining results revealed a significantly higher number of new blood vessels in the wounds treated with the agonist at 5-days post-injury (Total number of blood vessels, 31.96 > 23.28, respectively) compared to the controls (Figures 5A–C). However, there was no significant change in the size of these blood vessels (Figure 5D) at this time. At 7-days post-injury, a significant increase in the average size of the blood vessels was observed in the agonist-treated group compared to the controls (624.50 mm^2^ > 490.09 mm^2^, respectively) (Figures 5E,F,H), but no significant changed observed in blood vessels count (Figure 5G). These results indicate that the 5-HT1A receptor agonist accelerates the early proliferative phase of healing, as more new vessels are formed earlier on. At the 7-day time point, the number of blood vessels almost equalizes between the two groups. However, the size of the blood vessels in the agonist group are significantly larger than the ones formed in the vehicle-treated wounds, as they have had more time to mature since they were formed earlier on. Therefore, observations suggest that the 5-HT1A receptor agonist accelerates and promotes skin healing partly through the enhancement of neovascularization.

## Discussion

The regenerative role of serotonin has already been well-established in the liver (Lesurtel et al., 2006; Amini-Nik et al., 2018). The 5-HT1A receptor specifically has been identified as a key modulator in several other tissues, such as within the spinal motor neuron regeneration process in zebrafish (Barreiro-Iglesias et al., 2015). Abundant expression of the 5-HT1A receptor in human and murine skin keratinocytes and fibroblasts (Supplementary Figure S1; Slominski et al., 2003) compared to other serotonin receptors prompted us to further investigate the role this receptor during skin healing. As it favors regeneration in the liver, we hypothesized whether downregulation of this pathway dampens the process of healing or whether upregulation of it might change the skin healing process toward a more regenerative phenotype.

The 5-Ht1a receptor KO mice group showed delayed and inefficient wound healing, exemplified by a significantly larger wound area, poor re-epithelization, high cellularity, and poor neovascularization. Although ubiquitous knock out of this receptor may not reveal all the cell types that are predominantly affected by this receptor, our *in vitro* data supports the notion that the 5-HT1A receptor influences the course of skin healing partly through fibroblast migration, an essential component of skin healing. Deficient migration of fibroblasts, which are the main cellular component within the dermis, may suggest an underlying factor which contributes to improper wound healing. The presence of a high macrophage count (Figure 2D) further implies that there is prolonged inflammation in the 5-HT1A receptor KO mice, which may impair the wound healing process (Qian et al., 2016). Our data, at this level, cannot verify whether the deficient migration of fibroblasts into the wound bed is the cause of perpetuated inflammation or if knocking out 5-Ht1a receptor in macrophages contributed in the observed inflammation in the KO animals.

Nevertheless, this interplay has been documented in both directions where macrophages can enhance fibroblast migration (Dewor et al., 2007; Amini-Nik et al., 2014; Amini-Nik et al., 2018) as well. In normal tissue, the 5-HT1A receptor is also expressed in platelets, neutrophils, macrophages, and mast cells, all of which are components of the wound microenvironment (Shah and Amini-Nik, 2017). Wound healing is dependent on several cell types including keratinocytes, macrophages, activated fibroblasts, and endothelial cells. Therefore, future experiments using macrophage-specific 5-Ht1a receptor KO mice are warranted to further study the effects of this receptor on macrophage function in the context of wound healing and to verify whether this effect is bidirectional.

Macrophages have been previously shown to promote and enhance neovascularization through secretion of pro-neovascularization cytokines (Mahdavian Delavary et al., 2011; Yang et al., 2016). However, in our study, despite having a relative abundance of macrophages, the KO animals had poor neovascularization compared to the wild-type group. The fewer number of newly formed blood vessels in the granulation tissue of the 5-Htr1a KO animals suggests that this receptor might regulate wound healing partly through blood vessel formation. Previous studies suggested that serotonin serves as an angiokine (Balsa et al., 1998; Goldhar et al., 2005; Iwabayashi et al., 2012) in different models. Observing fewer blood vessels in KO animals, despite the higher number of macrophages in the wounds of these animals, further supports the notion that the 5-HT1A receptor might directly affect neovascularisation, independent of macrophages.

We described the enhancement of wound healing in mice by employing a 5-HTR1A agonist treatment. The 5-HTR1A agonist treatment (8-Hydroxy-DPAT hydrobromide) (Hiner et al., 1988; Sakai and Tanaka, 1993) resulted in significantly lower cellular density and smaller wound scar size as compared to the control (*in vivo*). It can be deduced that the 5-HT1A receptor agonist enhances fibroblast migration into the wound bed which creates a microenvironment that facilitates wound healing, leading to a smaller scar size with enhanced neovascularization. Furthermore, these results also suggest that minimizing excessive scarring at later stages can help to improve healing (Block et al., 2015). We demonstrated that the 5-HT1A receptor agonist treatment increased blood vessel count (CD31 expression) during the early proliferation phase and blood vessel size later in the proliferation phase. Neovascularization is a major contributing factor toward improved healing as new blood vessels provide more oxygen and nutrients which promote growth (Demidova-Rice et al., 2012; Park et al., 2016). During wound healing, platelets are recruited to the site and adhere to the activated vascular wall and release pro-angiogenic factors such as vascular endothelial growth factor A (VEGF-A) and serotonin (Peters et al., 2014). Serotonin is known to promote angiogenesis in endothelial cells through downstream activated Src/PI3K/AKT/mTOR/p70S6K phosphorylation signaling involving G-protein-coupled receptors (Zamani and Qu, 2012). Additionally, the previous study conducted in hepatocellular carcinoma patients also demonstrated that serotonin stimulation through the 5-HT1A receptor is associated with a higher micro-vessel density and VEGF expression (Zamani and Qu, 2012). These studies support our conclusions regarding the role of 5-HT1A receptor in promoting angiogenesis during wound healing.

Our study is limited by the non-specificity of serotonin receptors agonists such that 8-hydroxy-DPAT hydrobromide, which is commonly used as a 5-HTR1A agonist, is also shown to be a 5-HTR7 agonist (Hedlund et al., 2004). Ideally, we should also investigate the effects of 5-hydroxy-DPAT on 5-HT1A receptor KO mice *in vivo* to pinpoint the mechanism of action. However, due to the fact that our model of 5-Ht1a receptor KO has 5-Ht1a receptor ubiquitously absent in all cell types, this experiment would be inconclusive. Future experiments should imply cell-specific 5-Ht1a receptor KO models to evaluate the mechanism of action.

## Conclusion

Collectively, this study advances our understanding about the role of serotonin through its 5-HT1A receptor in cutaneous wound healing. There is impaired wound healing highlighted by a larger wound area, reduced fibroblast migration, and enhanced inflammation seen in 5-Ht1a receptor KO mice. Conversely, treatment with a topical cream formulation containing the 5-HT1A receptor agonist showed improvement in skin healing via accelerated neovascularization and fibroblast migration. We conclude that endogenous serotonin may have healing potential through 5-HT1A receptor-mediated downstream signaling cascades that regulate fibroblast migration in wounds. Future experiments using cell-specific 5-Ht1a receptor KO mice are warranted to evaluate the effects of this receptor on the functioning of different cell types in the context of wound healing.

## Author Contributions

AS performed the experiments, generated the data in Figures 1–4 and Supplementary Figure S1, and contributed in writing. IM contributed in performing the experiments, generated the data in Figure 5 and Supplementary Figure S2, and contributed in writing. AhS contributed to the analysis of the data and writing the manuscript. CB and WC-A assisted in experimental studies data analysis. MJ obtained funding and supervised the project. MH reviewed the manuscript. SA-N conceived the study, developed the hypothesis, designed the experiments, and reviewed the study. All authors discussed the results and contributed to the final manuscript.

## Conflict of Interest Statement

The authors declare that the research was conducted in the absence of any commercial or financial relationships that could be construed as a potential conflict of interest.

## Supplementary Material

FIGURE S1 Analysis of mRNA expression of serotonin receptors by RT-PCR. Expression of serotonin receptor 1A, 1B, 2A, 2B, and 7 in human keratinocytes **(A)** and human fibroblasts **(B)**. Expression of serotonin receptor 1A in human dermal skin tissue **(C)**, human keratinocytes and fibroblasts **(D)** and murine keratinocytes and fibroblasts **(E)**. Lane 1 represents 50 bp Ladder in each image. GAPDH was considered as control.

FIGURE S2 *In vivo* study design, effect of agonist on skin wound healing (excisional punch biopsy model).
