# Supplementary figures and images for: 5-HT1A Receptor Function Makes Wound Healing a Happier Process
Source: Front Pharmacol. 2018 Dec 11;9:1406. doi: 10.3389/fphar.2018.01406 (PMC6297675; doi:10.3389/fphar.2018.01406)

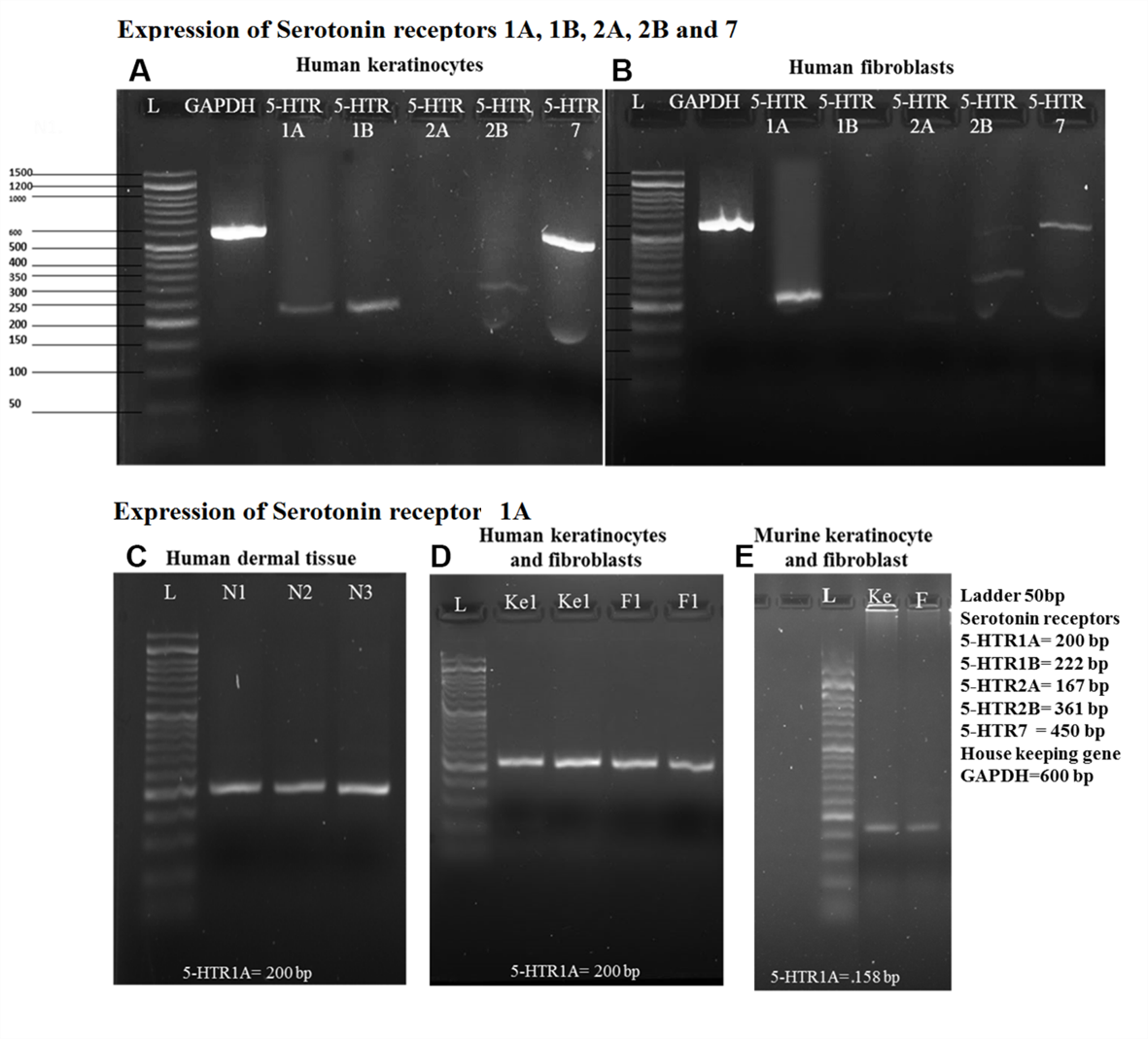

Supplement: FIGURE S1 — Analysis of mRNA expression of serotonin receptors by RT-PCR. Expression of serotonin receptor 1A, 1B, 2A, 2B, and 7 in human keratinocytes (A) and human fibroblasts (B). Expression of serotonin receptor 1A in human dermal skin tissue (C), human keratinocytes and fibroblasts (D) and murine keratinocytes and fibroblasts (E). Lane 1 represents 50 bp Ladder in each image. GAPDH was considered as control. [file Image_1.tif]

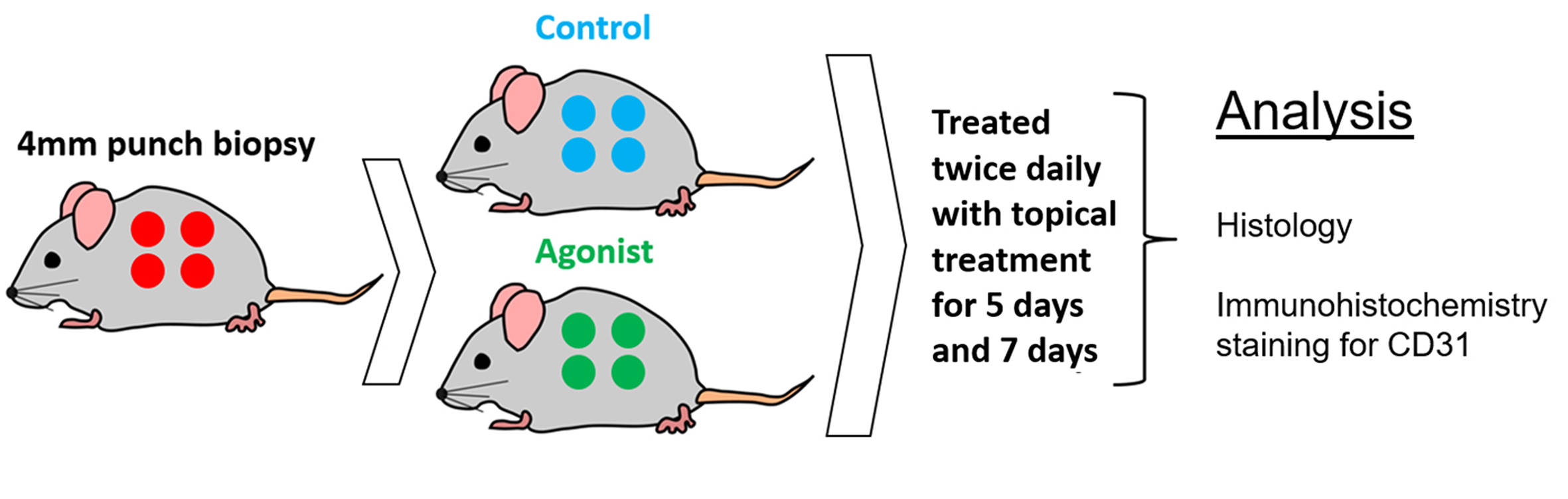

Supplement: FIGURE S2 — In vivo study design, effect of agonist on skin wound healing (excisional punch biopsy model). [file Image_2.TIF]
